# Narcissism and Anti-Immigrant Attitudes: A Tale of Pride and Prejudice?

**DOI:** 10.3390/bs15040451

**Published:** 2025-04-01

**Authors:** Virgil Zeigler-Hill, Angelina Toma, Emily Thomas, Avi Besser

**Affiliations:** 1 Department of Psychology, Oakland University, Rochester, MI 48309, USA; angelinatoma@oakland.edu (A.T.); em@stackcatcontent.com (E.T.); 2 Department of Communication Disorders, Jerusalem Multidisciplinary College, Jerusalem 9101001, Israel; avibe@jmc.ac.il

**Keywords:** narcissism, prejudice, worldviews, ideological attitudes, immigrant

## Abstract

Narcissism has been linked to negative attitudes toward certain outgroups. The present studies examined the associations that narcissistic traits—extraverted narcissism, antagonistic narcissism, and neurotic narcissism—had with anti-immigrant attitudes. More specifically, we were interested in the possibility that these associations may be mediated by social worldviews and ideological attitudes. Across three studies, the results indicated that extraverted and antagonistic narcissism had positive indirect associations with anti-immigrant attitudes through the competitive social worldview via the ideological attitudes of Right-Wing Authoritarianism and Social Dominance Orientation. In contrast, neurotic narcissism was negatively associated with anti-immigrant attitudes, though this relationship was not consistently mediated by social worldviews or ideological attitudes. These findings suggest that individuals with high levels of extraverted and antagonistic narcissism may endorse a competitive worldview, which aligns with negative attitudes toward immigrants who may be perceived as threats to their social status. This underscores the role of narcissism, social worldviews, and ideological attitudes in potentially shaping anti-immigrant sentiment.

## 1. Introduction

The rising prevalence of anti-immigrant attitudes in contemporary societies has prompted extensive research into the psychological foundations of these sentiments (Esses, 2021; Esses et al., 2001; Yakushko, 2009). Anti-immigrant attitudes encompass negative evaluations, biases, and prejudices toward individuals perceived as foreign or belonging to immigrant groups. Although socio-political and economic factors undoubtedly influence these attitudes, an increasing body of evidence highlights the role of personality traits in shaping intergroup biases (e.g., low openness and low agreeableness; Crawford & Brandt, 2019; Ekehammar & Akrami, 2007; Ekehammar et al., 2004; Hodson et al., 2009; Sibley & Duckitt, 2008). Among these traits, narcissism has emerged as a particularly relevant construct. The reason is that narcissism—which is characterized by self-centeredness, entitlement, and a lack of empathy for others—has been found to be linked with issues surrounding prejudice (Cichocka et al., 2017; Hodson et al., 2009; Mayer et al., 2020; Schnieders & Gore, 2011). This is likely because narcissistic individuals often perceive the social environment as inherently competitive, which can lead them to view outgroup members—including immigrants—as threats to the existing social structure. As a result, individuals with higher levels of narcissism may be more prone to endorsing exclusionary beliefs and discriminatory attitudes toward immigrants. The present research examines the connections that distinct narcissistic traits—extraverted narcissism, antagonistic narcissism, and neurotic narcissism—have with anti-immigrant attitudes. Specifically, we examine whether these associations are mediated by certain social worldviews and ideological attitudes.

### 1.1. Narcissistic Traits

Narcissism is a multifaceted personality construct defined as “entitled self-importance” (Krizan & Herlache, 2018, p. 6). It manifests through grandiosity, self-centeredness, feelings of superiority, and a willingness to exploit others (Morf & Rhodewalt, 2001). Building on the principle that personality traits are linked with individual differences in motivation (Denissen & Penke, 2008), theoretical frameworks suggest that narcissism may be fundamentally driven by the pursuit of status (Grapsas et al., 2020; Zeigler-Hill et al., 2018, 2019). This focus on status explains the competitive nature of narcissistic individuals, who seek to assert their dominance and demonstrate their superiority (Carter et al., 2015; Luchner et al., 2011; Morf et al., 2000). Narcissists often approach social interactions as competitions (Luchner et al., 2011), base their self-worth on outperforming others (Zeigler-Hill et al., 2008; Zeigler-Hill & Vrabel, 2023), and prioritize traits like competence and achievement over warmth and affiliation (Campbell et al., 2007).

There is a growing consensus that narcissism is not a monolithic construct. Instead, there is clear evidence that narcissism consists of multiple dimensions (Back et al., 2013; Krizan & Herlache, 2018). One model that has been used to describe the multidimensional structure of narcissism is the Trifurcated Model of Narcissism (TriMN; Crowe et al., 2019; Miller et al., 2016; Weiss et al., 2019) which delineates three dimensions: extraverted narcissism (characterized by exhibitionism and self-assurance), antagonistic narcissism (marked by defensiveness and aggression), and neurotic narcissism (typified by distress and a strong desire for approval). Although these narcissistic traits share core elements of self-absorption and distorted self-image, they differ in their interpersonal dynamics. Extraverted narcissism is marked by a desire to project superiority, antagonistic narcissism reflects competitive dominance, and neurotic narcissism is associated with vulnerability and insecurity (Crowe et al., 2019; Miller et al., 2017). The competitive nature of narcissism, which is most prominent in extraverted and antagonistic narcissism, drives individuals to view social interactions as zero-sum games, reinforcing hierarchical and dominance-oriented beliefs (Zeigler-Hill et al., 2018, 2019).

### 1.2. Social Worldviews and Ideological Attitudes

Social worldviews are cognitive frameworks that reflect individuals’ beliefs about the nature of their social environments, including expectations about others’ behaviors (Duckitt & Fisher, 2003). Once formed, social worldviews serve as anchors for individuals’ beliefs, predisposing them to favor information that aligns with their existing perspectives (Perry et al., 2013b; Sibley & Duckitt, 2008; Sibley et al., 2007). Importantly, social worldviews play a key role in shaping and maintaining attitudes and values, including those related to prejudice (Duckitt, 2001; Duckitt & Sibley, 2009; Sibley & Duckitt, 2008, 2013).

The Dual Process Cognitive-Motivational Model (Duckitt, 2001) provides a framework for understanding how social worldviews contribute to the formation of ideological attitudes, specifically Right-Wing Authoritarianism (RWA; Altemeyer, 1981) and Social Dominance Orientation (SDO; Pratto et al., 1994). RWA is characterized by a preference for traditional values, submission to authority, ethnocentrism, and hostility toward outgroups (Altemeyer, 1981). In contrast, SDO reflects a preference for hierarchical intergroup relations and the belief that one’s own group is superior to others (Pratto et al., 1994).

The Dual Process Cognitive-Motivational Model (Duckitt, 2001) builds on earlier motivated cognition theories, which argue that the connections between social worldviews and ideological attitudes are mediated by specific motives (Ross, 1993). According to this model, individuals with a dangerous social worldview—those who perceive the world as threatening and unpredictable—are more likely to develop higher levels of RWA (Duckitt et al., 2002; Perry et al., 2013b). The reason is that these perceptions of danger activate motives related to social cohesion, control, and collective security, which, in turn, foster the development of RWA as a means of achieving safety (Zeigler-Hill, 2019).

In contrast, individuals with a competitive social worldview—those who view the world as a cutthroat, Darwinian environment—tend to develop higher levels of SDO (Duckitt et al., 2002; Perry et al., 2013b; Sibley et al., 2007). The tendency to view the social environment as being hyper-competitive activates motives focused on dominance and superiority, which promote the development of SDO as a strategy for achieving these goals (Zeigler-Hill, 2019). This distinction is important because, despite similarities between RWA and SDO (e.g., both are positively associated with prejudicial attitudes; Duckitt, 2001), these ideological attitudes may stem from different worldviews and distinct social motives (Duckitt et al., 2002).

Narcissism has been linked to certain ideological attitudes (Bardeen & Michel, 2019; Cichocka et al., 2017; Hatemi & Fazekas, 2018; Hodson et al., 2009; Jonason, 2015; van Hiel & Brebels, 2011; Zeigler-Hill et al., 2021; see Cichocka et al., 2024, for a review). One potential explanation for these connections is that extraverted narcissism and antagonistic narcissism have been found to be associated with the competitive social worldview (Dehaghi & Zeigler-Hill, 2021; Zeigler-Hill et al., 2021). Research has shown that the competitive social worldview mediates the links these aspects of narcissism have with SDO (Zeigler-Hill et al., 2021). The tendency to adopt a competitive social worldview may help explain why narcissistic traits—especially antagonistic narcissism—tend to have stronger associations with SDO (which aligns with narcissistic motivations for status and power) than with RWA (which emphasizes conformity and collective security and may conflict with the narcissistic desire for individuality and self-expression; Cichocka et al., 2017; Golec de Zavala et al., 2013; Hart & Stekler, 2021; Hodson et al., 2009; Jonason, 2015; Mayer et al., 2020; Moor et al., 2019; Zeigler-Hill et al., 2021). Taken together, these results suggest that individuals with elevated levels of extraverted narcissism and antagonistic narcissism are likely to find SDO appealing.

### 1.3. Linking Narcissism to Anti-Immigrant Attitudes Through Worldviews and Ideological Attitudes

Individuals with high levels of narcissism are often preoccupied with issues related to status (Grapsas et al., 2020; Zeigler-Hill et al., 2018, 2019), which aligns with previous research suggesting that narcissism is strongly linked to a desire for dominance, power, and control over others (Bushman & Baumeister, 1998; Raskin et al., 1991; Zeigler-Hill & Dehaghi, 2023). As a result, individuals with high levels of narcissistic traits may be predisposed to adopt prejudicial attitudes toward outgroup members as a means of reinforcing their superior position within the social hierarchy. This is supported by numerous studies that have found positive associations between narcissism and various forms of prejudice (Anderson & Cheers, 2018; Bizumic & Duckitt, 2008; Hodson et al., 2009; Jonason, 2015; Jonason et al., 2020; Kay & Dimakis, 2024; Keiller, 2010; Koehn et al., 2019; Mayer et al., 2020; McFarland, 2010; Moor et al., 2019).

Although the associations between narcissism and prejudice tend to be small-to-medium in magnitude, they have emerged across samples from diverse countries (e.g., United States, New Zealand, Serbia, Australia) and have been observed for prejudice toward various outgroups (e.g., racial/ethnic minority groups, sexual minorities). However, some studies have reported weaker or unclear links between narcissism and prejudice. For instance, no association was found between narcissism and prejudice toward the Roma in an Italian sample (Colledani et al., 2018). In addition, narcissism was only associated with negative attitudes toward immigrants under conditions of perceived threat in college students from the United States (Schnieders & Gore, 2011).

Taken together, existing research suggests that narcissistic individuals may be particularly prone to adopting negative stereotypes or dehumanizing attitudes toward outgroup members when they perceive these groups as threatening their place in the social hierarchy. In this context, outgroup members—such as immigrants—may be viewed as obstacles to the fulfillment of narcissistic desires for status and admiration. This mindset can further fuel prejudiced attitudes, as individuals with elevated narcissism may interpret the presence of immigrants as a competitive threat to their social standing, reinforcing exclusionary behaviors and discriminatory beliefs.

To address the complex association between narcissism and prejudice, previous studies have sometimes included SDO and RWA as potential mediators of this link (Cichocka et al., 2017; Hodson et al., 2009; Żemojtel-Piotrowska et al., 2020). These studies suggest that narcissism often has positive indirect associations with prejudice through SDO, implying that narcissistic individuals’ desire for dominance may lead to greater prejudice, particularly toward outgroups. In contrast, these studies show weaker and less consistent associations between narcissism and prejudice through RWA, which emphasizes conformity and group cohesion.

A key limitation of previous research concerning the role that ideological attitudes play in the connections between narcissism and prejudice is the tendency for some of those studies to treat narcissism as a unitary construct, rather than recognizing it as being multidimensional. This issue extends to many studies examining the broader connection between narcissism and prejudice, which have often failed to address the multidimensional nature of narcissism. A more nuanced approach that considers the various aspects of narcissism could provide a clearer understanding of its connections with prejudice.

### 1.4. Overview and Predictions

Previous studies have examined the connections that narcissism has with anti-immigrant attitudes (Hodson et al., 2009; Mayer et al., 2020; Schnieders & Gore, 2011). However, none of those studies have considered how social worldviews and ideological attitudes might mediate the associations that extraverted narcissism, antagonistic narcissism, and neurotic narcissism have with anti-immigrant attitudes. Understanding these pathways may be essential for disentangling the complex psychological mechanisms underlying the connections between narcissism and anti-immigrant attitudes. The present studies address these gaps by examining the connections these narcissistic traits have with anti-immigrant attitudes as well as the extent to which these associations are mediated by social worldviews and ideological attitudes. Specifically, we developed the following hypotheses:

**Hypothesis 1.**  *We hypothesized that antagonistic narcissism would be positively associated with anti-immigrant attitudes. This hypothesis is grounded in the core features of antagonistic narcissism, such as hostility, suspiciousness, exploitation of others, and a lack of empathy (Weiss et al., 2019). These traits may predispose individuals to perceive outgroups, particularly immigrants, as potential competitors. The tendency for antagonistic narcissists to see others as inferior and untrustworthy could manifest in heightened prejudice toward immigrant groups, whom they may perceive as potential rivals for resources and status. Additionally, the characteristic aggressive defensiveness and sensitivity to perceived challenges associated with antagonistic narcissism (Vize et al., 2020) may trigger hostile reactions toward cultural outgroups seen as disrupting the existing social order. This prediction aligns with the results of a previous study showing that narcissistic rivalry—which is similar to antagonistic narcissism—was positively associated with anti-immigrant attitudes (Mayer et al., 2020).*

**Hypothesis 2.**  *We hypothesized that the positive association between antagonistic narcissism and anti-immigrant attitudes would be serially mediated by the competitive social worldview and SDO. Specifically, we expected that antagonistic narcissism would have a positive indirect association with SDO through the competitive social worldview, a prediction that aligns with previous research (Zeigler-Hill et al., 2021). Furthermore, we anticipated that this mediational pathway would extend to anti-immigrant attitudes, given the well-established connection between SDO and such attitudes in prior studies (Küpper et al., 2010; Mayer et al., 2020; Newman et al., 2014; Pellegrini, 2023; Wollast et al., 2023). In other words, we hypothesized that antagonistic narcissism would be positively related to the competitive social worldview, which would then be positively associated with SDO. Subsequently, SDO would be positively related to anti-immigrant attitudes. Although we did not have specific hypotheses regarding the indirect association that antagonistic narcissism would have with anti-immigrant attitudes through the dangerous social worldview or RWA, we included those constructs in our analyses for exploratory purposes. Previous studies have shown that both constructs are linked to prejudice (Duckitt & Sibley, 2009; Sibley & Duckitt, 2013; Sibley et al., 2007), making them relevant to consider in the context of our investigation.*

**Hypothesis 3.**  *We did not have clear predictions concerning whether extraverted narcissism would have direct or indirect associations with anti-immigrant attitudes. The reason for our uncertainty is that it appears that extraverted narcissism may have complex connections with anti-immigrant attitudes. On one hand, extraverted narcissism has been shown to have a positive indirect association with SDO through the competitive social worldview (Zeigler-Hill et al., 2021). This would suggest that extraverted narcissism may have an indirect association with anti-immigrant attitudes given that SDO has consistently been shown to be associated with anti-immigrant attitudes (Küpper et al., 2010; Mayer et al., 2020; Newman et al., 2014; Pellegrini, 2023; Wollast et al., 2023). However, Mayer et al. (2020) found that narcissistic admiration—which is similar to extraverted narcissism—was not correlated with anti-immigrant attitudes. Despite our lack of clear hypotheses concerning the potential connections between extraverted narcissism and anti-immigrant attitudes, we included this aspect of narcissism in our studies for exploratory purposes.*

**Hypothesis 4.**  *We did not have clear predictions concerning whether neurotic narcissism would have direct or indirect associations with anti-immigrant attitudes. The reason for our uncertainty is that the connection between neurotic narcissism is unclear. The vulnerability, insecurity, and emotional instability characteristic of neurotic narcissism might lead individuals to perceive immigrants as threatening to their fragile sense of self and social status, potentially fostering prejudicial attitudes. Additionally, the heightened anxiety and defensive self-protection associated with neurotic narcissism could manifest as fear of cultural change or economic instability attributed to immigration. However, the self-consciousness and social withdrawal typical of neurotic narcissists might also reduce direct competition with outgroups, potentially weakening anti-immigrant sentiment. Furthermore, the tendency toward rumination and self-focused distress in neurotic narcissism may lead these individuals to be less concerned with broader societal issues like immigration. Prior research has shown that the basic personality dimension of neuroticism—which has similarities to neurotic narcissism—has not been shown to have any consistent association with prejudice (Crawford & Brandt, 2019; Sibley & Duckitt, 2008), suggesting that neurotic narcissism is likely to have little, if any, association with anti-immigrant attitudes. Despite our lack of clear hypotheses, we included neurotic narcissism in our analyses for exploratory purposes.*

## 2. Study 1

The purpose of Study 1 was to examine whether narcissistic traits had indirect associations with anti-immigrant attitudes through social worldviews and ideological attitudes in a sample of undergraduate students from the United States.

### 2.1. Participants and Procedure

This study involved 442 undergraduate students from a university in the Midwestern region of the United States. Participants completed the study in exchange for partial fulfillment of a research participation requirement. Participants completed measures related to narcissism, social worldviews, ideological attitudes, and anti-immigrant attitudes via an online portal. Additional measures not relevant to the present study, such as those assessing motivation, were also included. These measures were presented in a random order that varied between participants.

Given the lack of established strategies for estimating the necessary sample size for serial parallel multiple mediation analyses, we conducted a power analysis for parallel indirect associations using Monte Carlo simulations (Schoemann et al., 2017). We assumed that the associations between the variables would be of medium-to-large magnitude. The power analysis indicated that a minimum sample size of 296 participants would be needed to achieve a power of at least 0.80 at α = 0.05. However, recognizing that this estimate did not account for the complexity of the serial mediation component of the model, we chose to oversample. Specifically, we implemented a time-based stopping rule, collecting data from as many participants as possible during the course of a single academic semester with the goal of obtaining an adequate sample size for testing our hypotheses.

Data from 76 participants were excluded due to inattentive or careless responses. More specifically, 45 participants were excluded for failing two or more directed response items designed to detect inattentiveness (e.g., “If you are reading this item, then please select ‘2’ as your response”), 9 were excluded as univariate outliers, 12 were excluded for inconsistent responding as indicated by high inter-item standard deviation (Marjanovic et al., 2015), and 10 were excluded for invariant response patterns identified through long-string analysis (Huang et al., 2012; Meade & Craig, 2012). The data were also screened for participants missing more than 5% of responses or identified as multivariate outliers, but no participants met these criteria for exclusion.

The final sample consisted of 366 participants (80 men, 274 women, 10 non-binary or gender-diverse individuals, and 2 individuals who preferred not to disclose their gender). The mean age of the final participants was 20.06 years (SD = 3.27), with ages ranging from 18 to 48 years. The racial and ethnic composition of the sample was 75% White, 9% Black, 5% Asian, 5% Hispanic, and 6% identifying as other.

### 2.2. Measures

#### 2.2.1. Narcissism

The short form of the Five-Factor Narcissism Inventory (Sherman et al., 2015) was used to capture extraverted narcissism (16 items; e.g., “I often fantasize about someday being famous” [α = 0.87]), antagonistic narcissism (32 items; e.g., “I’m pretty good at manipulating people” [α = 0.92]), and neurotic narcissism (12 items; e.g., “I often feel as if I need compliments from others in order to be sure of myself” [α = 0.88]). Participants were asked to rate their level of agreement with each statement using scales that ranged from 1 (disagree strongly) to 5 (agree strongly).

#### 2.2.2. Dangerous and Competitive Social Worldviews

The Social Worldviews Scale-Revised (Perry et al., 2013a) was used to capture the dangerous social worldview (10 items; e.g., “Any day now chaos and anarchy could erupt around us. All the signs are pointing to it” [α = 0.78]) and the competitive social worldview (10 items; e.g., “My knowledge and experience tells me that the social world we live in is basically a competitive ‘jungle’ in which the fittest survive and succeed, in which power, wealth, and winning are everything, and might is right” [α = 0.73]). Participants were asked to indicate their level of agreement with each item using scales that ranged from 1 (strongly disagree) to 7 (strongly agree).

#### 2.2.3. Right-Wing Authoritarianism

RWA was measured using the Authoritarianism-Conservatism-Traditionalism Scale (Duckitt et al., 2010), which is a 36-item instrument (e.g., “What our country needs most is discipline, with everyone following our leaders in unity” [α = 0.93]). Participants were asked to rate their level of agreement with each statement using scales that ranged from −4 (very strongly disagree) to +4 (very strongly agree).

#### 2.2.4. Social Dominance Orientation

SDO was measured using the SDO-7 Scale (Ho et al., 2015), which is a 16-item instrument (e.g., “It is unjust to try to make groups equal” [α = 0.92]). Participants were asked to rate their level of agreement with each statement using scales that ranged from 1 (strongly oppose) to 7 (strongly favor).

#### 2.2.5. Anti-Immigrant Attitudes

Anti-immigrant attitudes were measured using the Immigrant Posse Scale (Thomsen et al., 2008), which captures the willingness of respondents to engage in the socially sanctioned persecution of immigrants. The participants were asked to “imagine that someday in the future your government decides to outlaw immigrant organizations and requests all citizens to do their best to make sure that the law has a successful effect”, Participants were then asked to indicate their level of agreement with each of the following items using scales that ranged from 1 (strongly disagree) to 7 (strongly agree): (1) “I would tell my friends that it was a good law”, (2) “I would tell the police about any members of immigrant organizations that I knew”, (3) “I would help hunt down members of immigrant organizations and turn them over to the police”, (4) “I would participate in attacks on immigrant headquarters if supervised by the proper authorities”, (5) “I would support the use of physical violence to make members of immigrant organization reveal the identity of other immigrants”, and (6) “I would support the execution of immigrant leaders”, The internal consistency of the Immigrant Posse Scale was α = 0.94 for the present study.

### 2.3. Data Analysis

The data analytic strategy employed in the present studies was consistent across all analyses. To begin, we used Pearson product-moment correlations to examine the zero-order relationships between narcissistic personality features, social worldviews, ideological attitudes, and anti-immigrant attitudes. These initial correlations provided a foundational understanding of the connections among the variables.

Building on these zero-order correlations, we followed these zero-order correlations with a series of serial parallel multiple mediation analyses that were conducted using a custom model in the PROCESS macro developed by Hayes (2018) to examine whether narcissistic personality features had indirect associations with anti-immigrant attitudes through social worldviews and ideological attitudes. Extraverted narcissism, antagonistic narcissism, and neurotic narcissism were analyzed separately in order to mitigate issues related to multicollinearity, which can arise when highly correlated predictors are included in the same analysis (Lynam et al., 2006).

It is important to emphasize that although we conceptualized these relationships using mediation models, the cross-sectional and correlational nature of the data precludes any causal inferences. Our findings should be interpreted as patterns of association rather than evidence of causal relationships.

### 2.4. Results and Discussion

**Figure 1:**
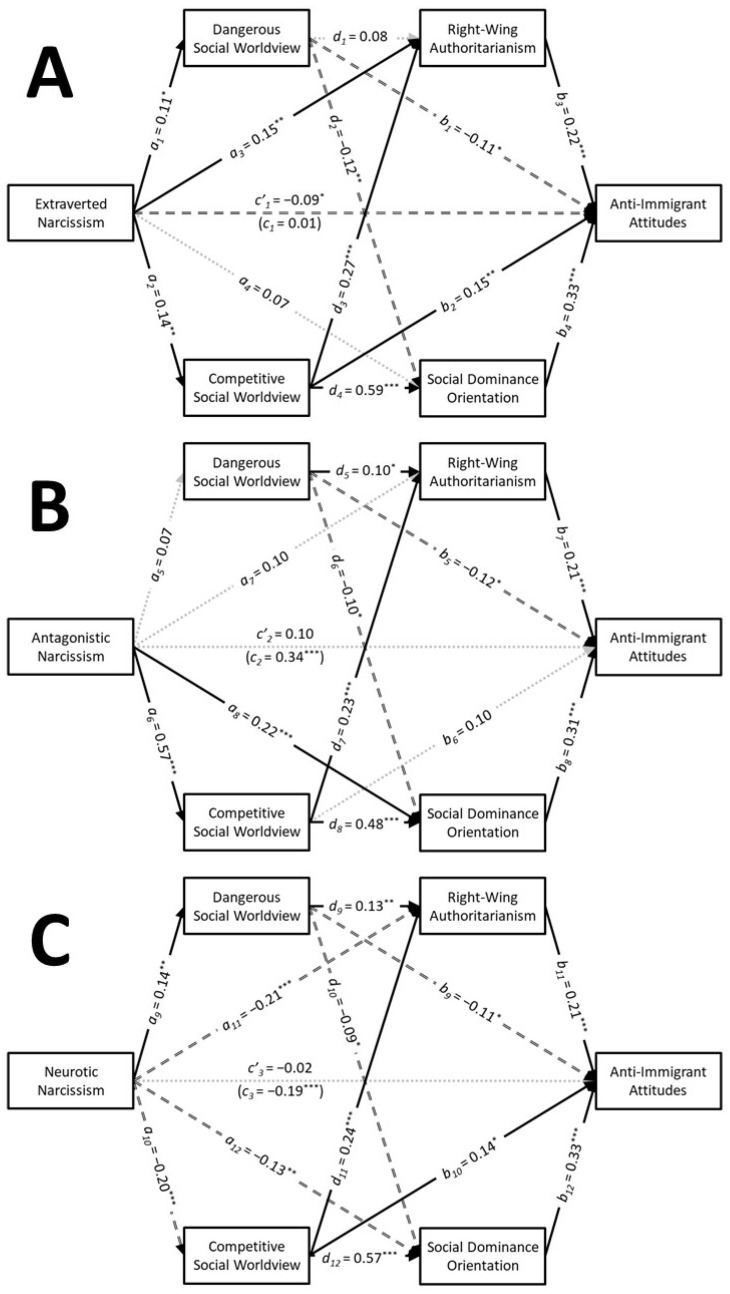
Study 1: The results of the serial parallel multiple mediation analyses showing the associations that extraverted narcissism (**A**), antagonistic narcissism (**B**), and neurotic narcissism (**C**) had with anti-immigrant attitudes through social worldviews via ideological attitudes. Note: Solid black arrows represent significant positive associations. Dashed black arrows represent significant negative associations. Dotted grey arrows represent nonsignificant associations. * *p* < 0.05; ** *p* < 0.01; *** *p* < 0.001.

Descriptive statistics and zero-order correlations are presented in Table 1. The results of the serial parallel multiple mediation analyses are depicted in Figure 1. These analyses revealed that extraverted narcissism had a significant positive association with the dangerous social worldview (a1 = 0.11, SE = 0.05, t = 2.09, *p* = 0.038, CI95% [0.01, 0.21], f2 = 0.01). However, this association was too weak to even be considered “small” in magnitude. Extraverted narcissism had small positive associations with the competitive social worldview (a2 = 0.14, SE = 0.05, t = 2.69, *p* = 0.008, CI95% [0.04, 0.24], f2 = 0.02) and RWA (a3 = 0.15, SE = 0.05, t = 2.91, *p* = 0.004, CI95% [0.05, 0.24], f2 = 0.02), but it was not linked with SDO (a4 = 0.07, SE = 0.04, t = 1.67, *p* = 0.095, CI95% [−0.01, 0.16], f2 = 0.01). Extraverted narcissism was found to have a positive indirect association with anti-immigrant attitudes through RWA (a3b3 = 0.03, SE = 0.01, z = 2.31, *p* = 0.021, CI95% [0.01, 0.06]). Tests of serial mediation indicated that extraverted narcissism also had positive indirect associations with anti-immigrant attitudes through the competitive social worldview via both RWA (a2d3b3 = 0.01, SE = 0.00, z = 2.07, *p* = 0.038, CI95% [0.00, 0.02]) and SDO (a2d4b4 = 0.03, SE = 0.01, z = 2.35, *p* = 0.019, CI95% [0.01, 0.05]). Although extraverted narcissism did not exhibit a significant total association with anti-immigrant attitudes (c1 = 0.01, SE = 0.05, t = 0.19, *p* = 0.847, CI95% [−0.09, 0.11], f2 = 0.00), it had a significant negative direct association when the mediators were included in the analysis (c’1 = −0.09, SE = 0.04, t = −2.00, *p* = 0.046, CI95% [−0.17, 0.00], f2 = 0.01). However, this association was too weak to even be considered “small” in magnitude. This analysis accounted for 34% of the variability in anti-immigrant attitudes.

Antagonistic narcissism exhibited a large positive association with the competitive social worldview (a6 = 0.57, SE = 0.04, t = 13.28, *p* < 0.001, CI95% [0.49, 0.66], f2 = 0.48) and a small positive association with SDO (a8 = 0.22, SE = 0.05, t = 4.36, *p* < 0.001, CI95% [0.12, 0.32], f2 = 0.05), but it was not associated with the dangerous social worldview (a5 = 0.07, SE = 0.05, t = 1.38, *p* = 0.169, CI95% [−0.03, 0.17], f2 = 0.01) or RWA (a7 = 0.10, SE = 0.06, t = 1.72, *p* = 0.086, CI95% [−0.01, 0.22], f2 = 0.01). Antagonistic narcissism was found to have a positive indirect association with anti-immigrant attitudes through SDO (a8b8 = 0.07, SE = 0.02, z = 3.15, *p* = 0.002, CI95% [0.03, 0.11]). Tests of serial mediation indicated that antagonistic narcissism also had positive indirect associations with anti-immigrant attitudes through the competitive social worldview via both RWA (a6d7b7 = 0.03, SE = 0.01, z = 2.65, *p* = 0.008, CI95% [0.01, 0.05]) and SDO (a6d8b8 = 0.08, SE = 0.02, z = 3.99, *p* < 0.001, CI95% [0.05, 0.13]). Although antagonistic narcissism had a small positive total association with anti-immigrant attitudes (c2 = 0.34, SE = 0.05, t = 7.01, *p* < 0.001, CI95% [0.25, 0.44], f2 = 0.13), this association did not persist when the mediators were included in the analysis (c’2 = 0.10, SE = 0.05, t = 1.79, *p* = 0.075, CI95% [−0.01, 0.20], f2 = 0.01). This analysis accounted for 33% of the variability in anti-immigrant attitudes.

Neurotic narcissism had a small positive association with the dangerous social worldview (a9 = 0.14, SE = 0.05, t = 2.71, *p* = 0.007, CI95% [0.04, 0.24], f2 = 0.02), whereas it had small negative associations with the competitive social worldview (a10 = −0.20, SE = 0.05, t = −3.94, *p* < 0.001, CI95% [−0.30, −0.10], f2 = 0.04), RWA (a11 = −0.21, SE = 0.05, t = −4.10, *p* < 0.001, CI95% [−0.31, −0.11], f2 = 0.05), and SDO (a12 = −0.13, SE = 0.04, t = −2.95, *p* = 0.003, CI95% [−0.21, −0.04], f2 = 0.02). Neurotic narcissism was found to have negative indirect associations with anti-immigrant attitudes through RWA (a11b11 = −0.04, SE = 0.01, z = −2.70, *p* = 0.007, CI95% [−0.07, −0.02]) and SDO (a12b12 = −0.04, SE = 0.02, z = −2.52, *p* = 0.012, CI95% [−0.08, −0.01]). Tests of serial mediation indicated that neurotic narcissism also had a negative indirect association with anti-immigrant attitudes through the competitive social worldview via SDO (a10d12b12 = −0.04, SE = 0.01, z = −3.04, *p* = 0.002, CI95% [−0.07, −0.02]). Although neurotic narcissism had a small negative total association with anti-immigrant attitudes (c3 = −0.19, SE = 0.05, t = −3.75, *p* < 0.001, CI95% [−0.29, −0.09], f2 = 0.04), this association did not persist when the mediators were included in the analysis (c’3 = −0.02, SE = 0.05, t = −0.33, *p* = 0.739, CI95% [−0.11, 0.08], f2 = 0.00). This analysis accounted for 33% of the variability in anti-immigrant attitudes.

These findings suggest distinct pathways through which narcissistic traits are associated with anti-immigrant attitudes. Extraverted narcissism did not have a total association with anti-immigrant attitudes, but it did have positive indirect associations with anti-immigrant attitudes through the competitive social worldview via both RWA and SDO. Despite the lack of a total association, extraverted narcissism had a small negative direct association with anti-immigrant attitudes when the mediators were included in the model. Antagonistic narcissism had a positive association with anti-immigrant attitudes (Hypothesis 1) as well as positive indirect associations, primarily through SDO, with serial mediation suggesting that the competitive social worldview played a role in this connection (Hypothesis 2). In contrast to the other narcissistic traits, neurotic narcissism showed negative indirect associations with anti-immigrant attitudes through the competitive social worldview via both RWA and SDO. The dangerous social worldview, however, did not serve as a significant mediator for any narcissistic trait, highlighting the importance of competitive dynamics in shaping these anti-immigrant attitudes. Collectively, these findings suggest that the indirect pathways linking narcissistic traits to anti-immigrant attitudes are mediated by the competitive social worldview and ideological attitudes, with variations across different facets of narcissism.

## 3. Study 2

The purpose of Study 2 was to replicate and extend the results of Study 1 by examining whether narcissistic traits had indirect associations with anti-immigrant attitudes through social worldviews and ideological attitudes in a sample of community members from the United States.

### 3.1. Method

#### 3.1.1. Participants and Procedure

This study involved 971 community members from the United States who were recruited through Prolific in exchange for financial compensation ($10). Participants completed measures of narcissism, social worldviews, ideological attitudes, and anti-immigrant attitudes—in addition to other instruments not relevant to the present study—via an online portal. Additional measures, unrelated to the present study, were also included to assess other constructs (e.g., spitefulness), and these measures were presented in a random order. As in Study 1, we initially estimated that a minimum of 296 participants would be required based on Monte Carlo simulations for parallel indirect associations. However, recognizing that this estimate did not account for the complexity of the serial mediation aspect of our model, we opted to over-sample. More specifically, we employed a financially based stopping rule, collecting data from as many participants as possible until the funds for the study were exhausted. This approach allowed us to ensure an adequate sample size for testing our hypotheses.

Data from 67 participants were excluded due to inattentive or careless responses using the same criteria as Study 1. More specifically, 20 participants were excluded for failing two or more attention checks, 14 were excluded for excessive amounts of missing data, 6 were excluded as univariate outliers, 5 participants were excluded for being multivariate outliers, 10 were excluded for inconsistent responding, and 12 were excluded for invariant response patterns.

The final sample consisted of 904 participants (469 men, 430 women, 3 non-binary or gender-diverse individuals, and 2 individuals who preferred not to disclose their gender). The mean age of the final participants was 32.61 years (SD = 9.92), with ages ranging from 18 to 77 years. The racial and ethnic composition of the sample was 69% White, 13% Black, 8% Asian, 4% Hispanic, and 6% identifying as other.

#### 3.1.2. Measures

Narcissism. Extraverted narcissism (α = 0.89), antagonistic narcissism (α = 0.95), and neurotic narcissism (α = 0.84) were measured using the Five-Factor Narcissism Inventory as in Study 1.

Dangerous and Competitive Social Worldviews. The dangerous social worldview (α = 0.80) and the competitive social worldview (α = 0.76) were measured using the Social Worldviews Scale-Revised as in Study 1.

Right-Wing Authoritarianism. RWA was measured using the Authoritarianism-Conservatism-Traditionalism Scale (α = 0.95) as in Study 1.

Social Dominance Orientation. SDO was measured using the SDO-7 Scale (α = 0.92) as in Study 1.

Anti-Immigrant Attitudes. Anti-immigrant attitudes were measured using the Immigrant Posse Scale (α = 0.96) as in Study 1.

#### 3.1.3. Results and Discussion

**Figure 2:**
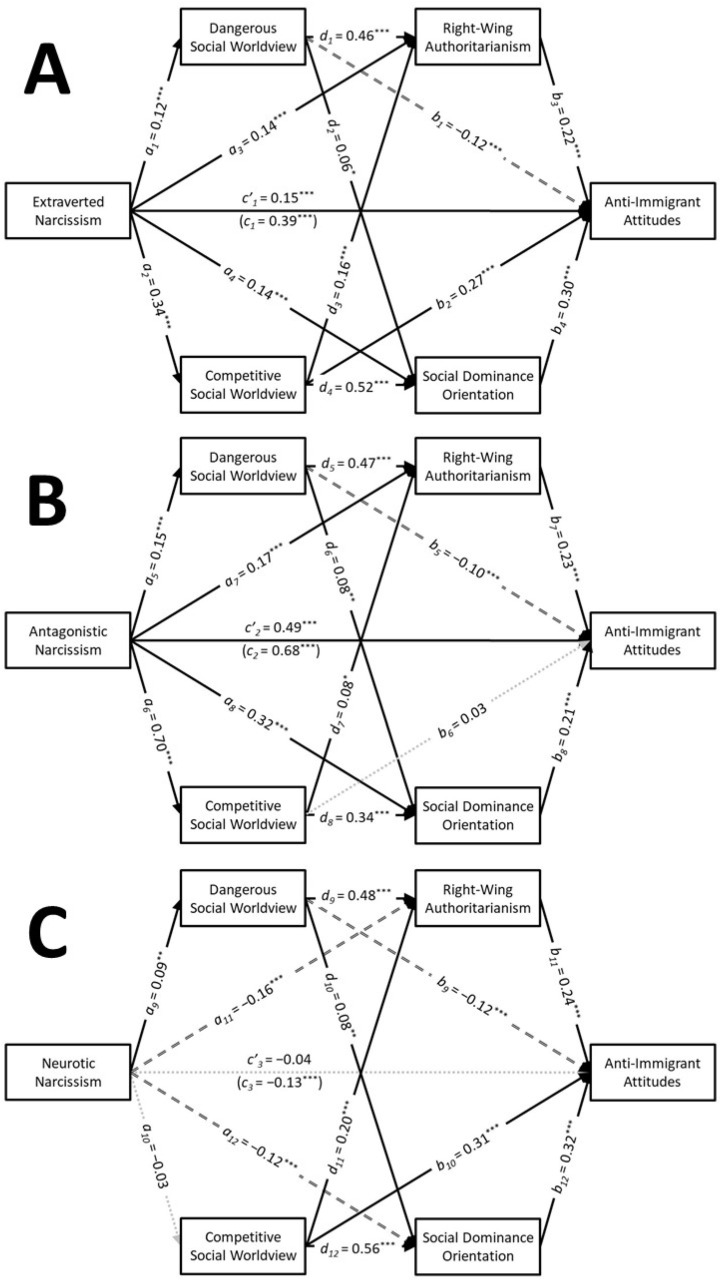
Study 2: The results of the serial parallel multiple mediation analyses showing the associations that extraverted narcissism (**A**), antagonistic narcissism (**B**), and neurotic narcissism (**C**) had with anti-immigrant attitudes through social worldviews via ideological attitudes. Note: Solid black arrows represent significant positive associations. Dashed black arrows represent significant negative associations. Dotted grey arrows represent nonsignificant associations. * *p* < 0.05; ** *p* < 0.01; *** *p* < 0.001.

Descriptive statistics and zero-order correlations are presented in Table 2. The results of the serial parallel multiple mediation analyses are depicted in Figure 2. These analyses revealed that extraverted narcissism had small positive associations with the dangerous social worldview (a1 = 0.12, SE = 0.03, t = 3.71, *p* < 0.001, CI95% [0.06, 0.19], f2 = 0.02), the competitive social worldview (a2 = 0.34, SE = 0.03, t = 10.88, *p* < 0.001, CI95% [0.28, 0.40], f2 = 0.13), RWA (a3 = 0.14, SE = 0.03, t = 4.92, *p* < 0.001, CI95% [0.09, 0.20], f2 = 0.03), and SDO (a4 = 0.14, SE = 0.03, t = 4.98, *p* < 0.001, CI95% [0.09, 0.20], f2 = 0.03). Extraverted narcissism was found to have positive indirect associations with anti-immigrant attitudes through the competitive social worldview (a2b2 = 0.09, SE = 0.01, z = 6.75, *p* < 0.001, CI95% [0.07, 0.12]), RWA (a3b3 = 0.03, SE = 0.01, z = 3.98, *p* < 0.001, CI95% [0.02, 0.05]), and SDO (a4b4 = 0.04, SE = 0.01, z = 4.34, *p* < 0.001, CI95% [0.02, 0.06]). Tests of serial mediation indicated that extraverted narcissism also had positive indirect associations with anti-immigrant attitudes through the competitive social worldview via both RWA (a2d3b3 = 0.01, SE = 0.00, z = 3.89, *p* < 0.001, CI95% [0.01, 0.02]) and SDO (a2d4b4 = 0.05, SE = 0.01, z = 6.49, *p* < 0.001, CI95% [0.04, 0.07]). Extraverted narcissism had a medium positive total association with anti-immigrant attitudes (c1 = 0.39, SE = 0.03, t = 12.58, *p* < 0.001, CI95% [0.33, 0.45], f2 = 0.17) that persisted when the mediators were included in the analysis (c’1 = 0.15, SE = 0.03, t = 5.83, *p* < 0.001, CI95% [0.10, 0.21], f2 = 0.04). This analysis accounted for 47% of the variability in anti-immigrant attitudes.

Antagonistic narcissism exhibited a large positive association with the competitive social worldview (a6 = 0.70, SE = 0.02, t = 29.37, *p* < 0.001, CI95% [0.65, 0.75], f2 = 0.96) as well as small positive associations with the dangerous social worldview (a5 = 0.15, SE = 0.03, t = 4.44, *p* < 0.001, CI95% [0.08, 0.21], f2 = 0.02), RWA (a7 = 0.17, SE = 0.04, t = 4.47, *p* < 0.001, CI95% [0.10, 0.25], f2 = 0.02), and SDO (a8 = 0.32, SE = 0.04, t = 8.95, *p* < 0.001, CI95% [0.25, 0.39], f2 = 0.09). Antagonistic narcissism was found to have positive indirect associations with anti-immigrant attitudes through RWA (a7b7 = 0.04, SE = 0.01, z = 3.86, *p* < 0.001, CI95% [0.02, 0.06]) and SDO (a8b8 = 0.07, SE = 0.01, z = 5.28, *p* < 0.001, CI95% [0.04, 0.09]). Tests of serial mediation indicated that antagonistic narcissism also had positive indirect associations with anti-immigrant attitudes through the competitive social worldview via SDO (a6d8b8 = 0.05, SE = 0.01, z = 5.26, *p* < 0.001, CI95% [0.03, 0.07]). Antagonistic narcissism had a large positive total association with anti-immigrant attitudes (c2 = 0.68, SE = 0.02, t = 28.04, *p* < 0.001, CI95% [0.63, 0.73], f2 = 0.87) that persisted when the mediators were included in the analysis (c’2 = 0.49, SE = 0.03, t = 15.19, *p* < 0.001, CI95% [0.43, 0.55], f2 = 0.26). This analysis accounted for 56% of the variability in anti-immigrant attitudes.

Neurotic narcissism had a significant positive association with the dangerous social worldview (a9 = 0.09, SE = 0.03, t = 2.81, *p* = 0.005, CI95% [0.03, 0.16], f2 = 0.01), but this association was too weak to even be considered a small effect size. In contrast, neurotic narcissism was not associated with the competitive social worldview (a10 = −0.03, SE = 0.03, t = −0.77, *p* = 0.441, CI95% [−0.09, 0.04], f2 = 0.00). Neurotic narcissism exhibited small negative associations with both RWA (a11 = −0.16, SE = 0.03, t = −5.93, *p* < 0.001, CI95% [−0.22, −0.11], f2 = 0.04) and SDO (a12 = −0.12, SE = 0.03, t = −4.37, *p* < 0.001, CI95% [−0.17, −0.06], f2 = 0.02). Neurotic narcissism was found to have negative indirect associations with anti−immigrant attitudes through RWA (a11b11 = −0.04, SE = 0.01, z = −4.51, *p* < 0.001, CI95% [−0.06, −0.02]) and SDO (a12b12 = −0.04, SE = 0.01, z = −3.95, *p* < 0.001, CI95% [−0.06, −0.02]). Although neurotic narcissism had a small negative total association with anti-immigrant attitudes (c3 = −0.13, SE = 0.03, t = −3.97, *p* < 0.001, CI95% [−0.20, −0.07], f2 = 0.02), this association did not persist when the mediators were included in the analysis (c’3 = −0.04, SE = 0.03, t = −1.71, *p* = 0.088, CI95% [−0.09, 0.01], f2 = 0.00). This analysis accounted for 45% of the variability in anti-immigrant attitudes.

These results largely mirror the findings of Study 1, with distinct patterns emerging for the three types of narcissism. Extraverted narcissism had positive indirect associations with anti-immigrant attitudes through the competitive social worldview via both RWA and SDO. Antagonistic narcissism had the expected positive association with anti-immigrant attitudes (Hypothesis 1) and indirect association with anti-immigrant attitudes through the competitive social worldview via SDO (Hypothesis 2). Antagonistic narcissism also had an unexpected positive indirect association with anti-immigrant attitudes through RWA. In contrast to the other narcissistic traits, neurotic narcissism had negative indirect associations with anti-immigrant attitudes via both RWA and SDO. These findings show that extraverted and antagonistic narcissism have positive associations with anti-immigrant attitudes through the competitive social worldview and ideological attitudes, whereas neurotic narcissism has negative associations with anti-immigrant attitudes through ideological attitudes. As in Study 1, the dangerous social worldview did not serve as a mediator for any narcissistic trait, highlighting the importance of competitive dynamics in shaping these anti-immigrant attitudes.

## 4. Study 3

The purpose of Study 3 was to replicate and extend the results of the previous studies by examining whether narcissistic traits had indirect associations with anti-immigrant attitudes through social worldviews and ideological attitudes in a sample of community members from Israel.

### 4.1. Method

#### 4.1.1. Participants and Procedure

This study involved 884 community members from Israel who volunteered by responding to recruitment requests distributed through flyers in public areas and postings on various social media platforms. Participants completed online surveys assessing narcissism, social worldviews, ideological attitudes, and anti-immigrant attitudes—in addition to other instruments not relevant to the present study—via an online portal. The questionnaires, originally in English, were translated into Hebrew using the back-translation method. Additional measures unrelated to the current study, such as those assessing other personality traits, were also included, and all measures were presented in a random order.

As in Studies 1 and 2, we estimated that a minimum of 296 participants would be required based on Monte Carlo simulations for parallel indirect associations. However, recognizing that this estimate did not account for the complexity of the serial mediation model, we opted to over-sample. More specifically, we employed a time-based stopping rule, collecting data from as many participants as possible during the course of a single academic year. This approach allowed us to ensure an adequate sample size for testing our hypotheses.

Data from 130 participants were excluded using the same predetermined criteria as the previous studies. More specifically, 74 participants were excluded for failing two or more attention checks, 42 were excluded as univariate outliers, 3 participants were excluded for being multivariate outliers, 6 were excluded for inconsistent responding, and 5 were excluded for invariant response patterns. The final sample consisted of 754 participants (333 men, 421 women). The mean age of the final participants was 30.09 years (SD = 9.22), with ages ranging from 18 to 66 years.

#### 4.1.2. Measures

Narcissism. Extraverted narcissism (α = 0.81), antagonistic narcissism (α = 0.83), and neurotic narcissism (α = 0.82) were measured using the Five-Factor Narcissism Inventory as in the previous studies.

Dangerous and Competitive Social Worldviews. The dangerous social worldview (α = 0.74) and the competitive social worldview (α = 0.67) were measured using the Social Worldviews Scale-Revised as in the previous studies.

Right-Wing Authoritarianism. RWA was measured using the Authoritarianism-Conservatism-Traditionalism Scale (α = 0.91) as in the previous studies.

Social Dominance Orientation. SDO was measured using the SDO-7 Scale (α = 0.84) as in the previous studies.

Anti-Immigrant Attitudes. Anti-immigrant attitudes were measured using the Immigrant Posse Scale (α = 0.85) as in the previous studies.

#### 4.1.3. Results

**Figure 3:**
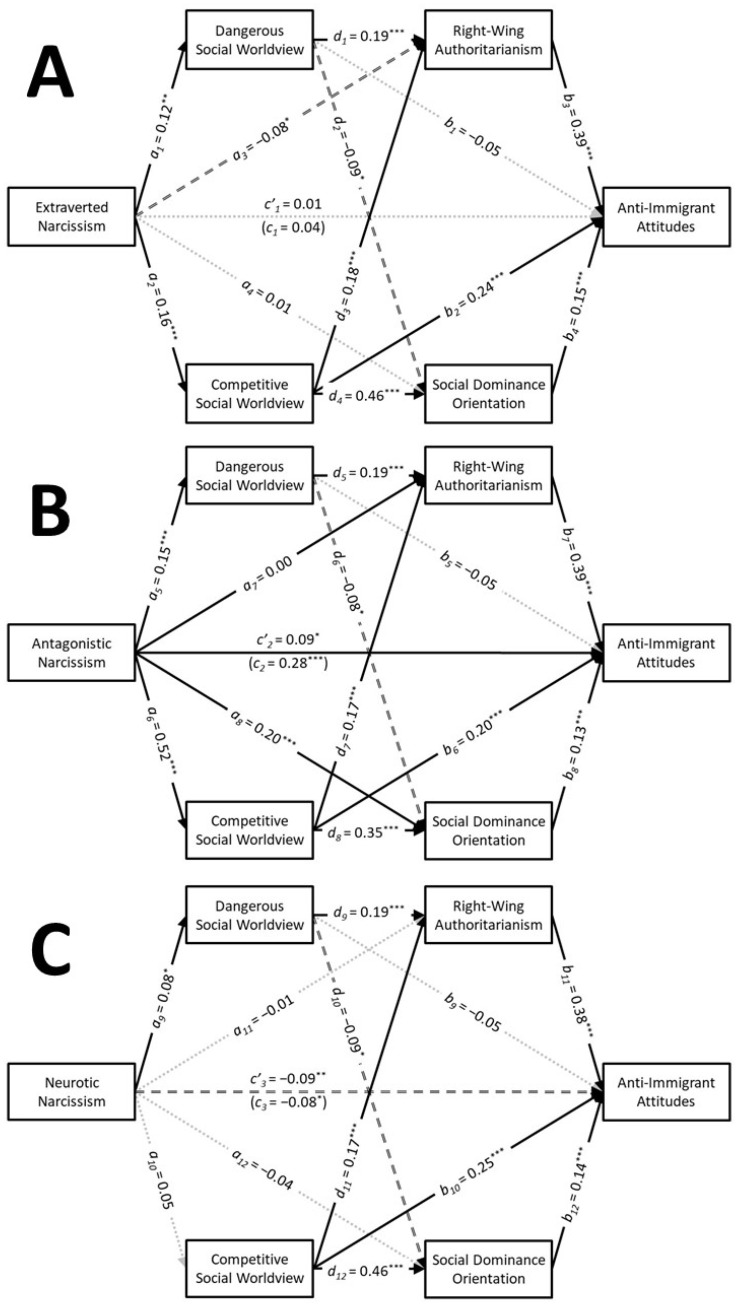
Study 3: The results of the serial parallel multiple mediation analyses showing the associations that extraverted narcissism (**A**), antagonistic narcissism (**B**), and neurotic narcissism (**C**) had with anti-immigrant attitudes through social worldviews via ideological attitudes. Note: Solid black arrows represent significant positive associations. Dashed black arrows represent significant negative associations. Dotted grey arrows represent nonsignificant associations. * *p* < 0.05; ** *p* < 0.01; *** *p* < 0.001.

Descriptive statistics and zero-order correlations are presented in Table 3. The results of the serial parallel multiple mediation analyses are depicted in Figure 3. These analyses revealed that extraverted narcissism had small positive associations with the dangerous social worldview (a1 = 0.12, SE = 0.04, t = 3.26, *p* = 0.001, CI95% [0.05, 0.19], f2 = 0.02) and the competitive social worldview (a2 = 0.16, SE = 0.04, t = 4.54, *p* < 0.001, CI95% [0.09, 0.23], f2 = 0.03), whereas it had a small negative association with RWA (a3 = −0.08, SE = 0.04, t = −2.19, *p* = 0.029, CI95% [−0.15, −0.01], f2 = 0.01) that was too weak to even be considered a “small” effect size. Extraverted narcissism was not associated with SDO (a4 = 0.01, SE = 0.03, t = 0.29, *p* = 0.769, CI95% [−0.06, 0.08], f2 = 0.00). Extraverted narcissism was found to have positive indirect associations with anti-immigrant attitudes through the competitive social worldview (a2b2 = 0.04, SE = 0.01, z = 3.69, *p* < 0.001, CI95% [0.02, 0.06]). Tests of serial mediation indicated that extraverted narcissism also had positive indirect associations with anti-immigrant attitudes through the competitive social worldview via both RWA (a2d3b3 = 0.01, SE = 0.00, z = 3.12, *p* = 0.002, CI95% [0.00, 0.02]) and SDO (a2d4b4 = 0.01, SE = 0.00, z = 3.02, *p* = 0.003, CI95% [0.00, 0.02]). Extraverted narcissism did not have a significant total association with anti-immigrant attitudes (c1 = 0.04, SE = 0.04, t = 1.23, *p* = 0.220, CI95% [−0.03, 0.12], f2 = 0.00) nor did an association emerge when the mediators were included in the analysis (c’1 = 0.01, SE = 0.03, t = 0.36, *p* = 0.720, CI95% [−0.05, 0.07], f2 = 0.00). This analysis accounted for 31% of the variability in anti-immigrant attitudes.

Antagonistic narcissism exhibited a large positive association with the competitive social worldview (a6 = 0.52, SE = 0.03, t = 16.54, *p* < 0.001, CI95% [0.46, 0.58], f2 = 0.36) as well as small positive associations with the dangerous social worldview (a5 = 0.15, SE = 0.04, t = 4.20, *p* < 0.001, CI95% [0.08, 0.22], f2 = 0.02) and SDO (a8 = 0.20, SE = 0.04, t = 5.20, *p* < 0.001, CI95% [0.12, 0.27], f2 = 0.04). In contrast, antagonistic narcissism was not associated with RWA (a7 = 0.00, SE = 0.04, t = −0.08, *p* = 0.936, CI95% [−0.08, 0.08], f2 = 0.00). Antagonistic narcissism was found to have positive indirect associations with anti-immigrant attitudes through the competitive social worldview (a6b6 = 0.10, SE = 0.02, z = 4.75, *p* < 0.001, CI95% [0.06, 0.15]) and SDO (a8b8 = 0.03, SE = 0.01, z = 3.00, *p* = 0.003, CI95% [0.01, 0.04]). Tests of serial mediation indicated that antagonistic narcissism also had positive indirect associations with anti-immigrant attitudes through the competitive social worldview via RWA (a6d7b7 = 0.03, SE = 0.01, z = 3.53, *p* < 0.001, CI95% [0.02, 0.05]) and SDO (a6d8b8 = 0.02, SE = 0.01, z = 3.36, *p* < 0.001, CI95% [0.01, 0.04]). Antagonistic narcissism had a small positive total association with anti-immigrant attitudes (c2 = 0.28, SE = 0.04, t = 7.96, *p* < 0.001, CI95% [0.21, 0.35], f2 = 0.08) that persisted when the mediators were included in the analysis (c’2 = 0.09, SE = 0.04, t = 2.57, *p* = 0.010, CI95% [0.02, 0.16], f2 = 0.01). However, the positive association between antagonistic narcissism and anti-immigrant attitudes was too weak to even be considered “small” in magnitude when the mediators were included in the model. This analysis accounted for 32% of the variability in anti-immigrant attitudes.

Neurotic narcissism had a significant positive association with the dangerous social worldview (a9 = 0.08, SE = 0.04, t = 2.23, *p* = 0.026, CI95% [0.01, 0.15], f2 = 0.01), but this association was too weak to even be considered a small effect size. Neurotic narcissism was not associated with the competitive social worldview (a10 = 0.05, SE = 0.04, t = 1.32, *p* = 0.187, CI95% [−0.02, 0.12], f2 = 0.00), RWA (a11 = −0.01, SE = 0.04, t = −0.25, *p* = 0.803, CI95% [−0.08, 0.06], f2 = 0.00), or SDO (a12 = −0.04, SE = 0.03, t = −1.21, *p* = 0.227, CI95% [−0.10, 0.02], f2 = 0.00). Neurotic narcissism did not have any significant indirect associations with anti-immigrant attitudes. Neurotic narcissism had a significant negative total association with anti-immigrant attitudes (c3 = −0.08, SE = 0.04, t = −2.10, *p* = 0.036, CI95% [−0.15, −0.01], f2 = 0.01) that persisted when the mediators were included in the analysis (c’3 = −0.09, SE = 0.03, t = −2.85, *p* = 0.004, CI95% [−0.15, −0.03], f2 = 0.01). However, the negative association between neurotic narcissism and anti-immigrant attitudes was too weak to even be considered “small” in magnitude, regardless of whether the mediators were included in the model. This analysis accounted for 32% of the variability in anti-immigrant attitudes.

The results from Study 3 largely replicated the patterns observed in the previous studies, with narcissistic personality features showing different indirect associations with anti-immigrant attitudes through social worldviews and ideological attitudes. Extraverted narcissism had positive indirect associations with anti-immigrant attitudes through the competitive social worldview via RWA and SDO. Antagonistic narcissism had a positive association with anti-immigrant attitudes (Hypothesis 1) as well as a positive indirect association through the competitive social worldview via SDO (Hypothesis 2). Antagonistic narcissism also had an unexpected positive indirect association with anti-immigrant attitudes through the competitive social worldview via RWA. Neurotic narcissism, in contrast, did not have any significant indirect associations with anti-immigrant attitudes through the social worldviews or ideological attitudes. However, it did have a small negative total association with anti-immigrant attitudes that persisted after including the mediators. Overall, the results suggest that extraverted and antagonistic narcissism have similar indirect association with anti-immigrant attitudes through the competitive social worldview via the ideological attitudes of RWA and SDO, whereas neurotic narcissism was negatively associated with anti-immigrant attitudes that did not operate through social worldviews or ideological attitudes. As in the previous studies, the dangerous social worldview did not serve as a significant mediator for the connections that the narcissistic trait had with anti-immigrant attitudes. One limitation of this study that should be noted is that the internal consistency for the competitive social worldview was relatively low.

## 5. General Discussion

The current research sought to examine the complex associations that narcissistic traits had with anti-immigrant attitudes, focusing on the roles of social worldviews and ideological attitudes as potential mediators. Across three studies, we tested a series of serial parallel multiple mediation models to understand whether different dimensions of narcissism—extraverted, antagonistic, and neurotic—were indirectly associated with anti-immigrant attitudes through the lenses of social worldviews (i.e., dangerous and competitive) and ideological attitudes (i.e., RWA and SDO). The results from all three studies largely supported our hypotheses, with each narcissistic trait showing distinct associations with the mediators and anti-immigrant attitudes. These findings contribute to the growing body of research that links narcissism to prejudice and intolerance, offering nuanced insights into the mechanisms underlying these associations.

The zero-order correlations between extraverted narcissism and anti-immigrant attitudes varied across studies, with a positive correlation found in Study 2 but not in Studies 1 or 3. However, extraverted narcissism consistently showed positive indirect associations with anti-immigrant attitudes through a competitive social worldview, via both RWA and SDO, across all three studies. Although we did not have specific predictions for extraverted narcissism, the results suggest that individuals with high levels of extraverted narcissism may be more likely to adopt a competitive social worldview, which, in turn, promotes the development of RWA and SDO. These ideological attitudes may then contribute to the emergence of negative attitudes toward outgroups, such as immigrants.

In support of Hypothesis 1, antagonistic narcissism showed consistent positive zero-order correlations with anti-immigrant attitudes across all three studies. Hypothesis 2 was also strongly supported, as antagonistic narcissism was found to have an indirect association with anti-immigrant attitudes through the competitive social worldview via SDO in each study. These findings suggest that individuals with high levels of antagonistic narcissism may develop anti-immigrant attitudes because they perceive immigrants as a threat to their own status or as being inferior to themselves, fostering disdain and prejudice. This mindset likely leads them to reject or belittle immigrants, viewing them as “outsiders” or potential competitors.

These results align with the notion that individuals high in antagonistic narcissism are more likely to hold prejudiced views toward outgroup members, as their worldviews emphasize competition and the endorsement of hierarchical structures that support dominance over outgroups. Previous research has linked antagonistic aspects of narcissism to prejudice (Mayer et al., 2020), and our findings extend this by demonstrating how this trait may be connected with anti-immigrant attitudes through worldviews and ideological beliefs.

Although we did not expect antagonistic narcissism to have indirect associations with anti-immigrant attitudes through RWA, such associations emerged in each study. In Study 2, RWA mediated the connection between antagonistic narcissism and anti-immigrant attitudes, while in the other studies, RWA played a role in serial mediation alongside the competitive social worldview. This suggests that antagonistic narcissism may be linked to anti-immigrant attitudes through pathways beyond maintaining the existing status hierarchy. Specifically, the indirect association through RWA implies that antagonistic narcissism may be connected to anti-immigrant attitudes due to concerns that immigrants could threaten cultural stability and social order. These beliefs, in turn, can foster prejudice and resistance to immigration as a challenge to the status quo (e.g., Armenta et al., 2023; Zárate et al., 2012).

Neurotic narcissism differed from other narcissistic traits in that it showed consistent negative associations with anti-immigrant attitudes across studies. In the two American samples (Studies 1 and 2), neurotic narcissism was negatively associated with anti-immigrant attitudes through RWA and SDO, suggesting that although individuals with neurotic narcissism may experience anxiety and insecurity, these feelings do not translate into the intergroup hostility seen in individuals with higher levels of extraverted or antagonistic narcissism. In fact, these findings indicate that neurotic narcissism may act as a protective factor against anti-immigrant biases. This pattern underscores the importance of distinguishing between different narcissistic traits when predicting social attitudes, such as those toward immigrants.

However, it is important to note that the negative indirect associations between neurotic narcissism and anti-immigrant attitudes via RWA and SDO did not emerge in the Israeli sample (Study 3). This discrepancy may reflect cultural differences in how neurotic narcissism relates to ideological attitudes—neurotic narcissism was negatively associated with RWA and SDO in the American samples but not in the Israeli sample.

The results of the present studies build on existing literature by clarifying the mechanisms that may help explain the connections between narcissistic traits and anti-immigrant attitudes. Previous studies have suggested that narcissism may be related to prejudice, but the roles of social worldviews and ideological attitudes as mediators have received little attention. Our findings provide evidence that social worldviews and ideological attitudes may be central to understanding the link between narcissism and anti-immigrant sentiment. For instance, the competitive worldview, which emphasizes group competition and dominance, appeared to be a key pathway through which extraverted and antagonistic narcissism were linked with anti-immigrant attitudes. These findings align with social dominance theory (Sidanius & Pratto, 1999), which posits that individuals who value group hierarchies are more likely to endorse prejudiced beliefs. Additionally, the role of RWA and SDO as mediators supports the notion that narcissistic individuals are more likely to endorse authoritarian and hierarchical ideologies, which, in turn, foster negative attitudes toward outgroups.

Despite the strengths of this research, several limitations must be acknowledged. First, the reliance on cross-sectional data in all three studies limits our ability to draw causal conclusions. Although the serial mediation models suggest that social worldviews and ideological attitudes mediate the relationship between narcissistic traits and anti-immigrant attitudes, these pathways cannot be definitively established as causal without longitudinal or experimental designs. Future research should aim to examine these relationships over time or in controlled experimental settings to confirm the directionality of the associations. Second, although we considered three distinct narcissistic traits, future research could further explore other individual differences that might also play a role in shaping anti-immigrant attitudes. For example, it would be valuable to investigate how characteristics such as socioeconomic status interact with narcissism to influence prejudiced beliefs. Third, the samples used in these studies were from the United States and Israel, which may limit the generalizability of the findings to other cultural groups. Future research should examine these relationships in other cultures to determine whether the patterns observed here hold across different contexts and populations. Fourth, the samples were predominantly White (e.g., 75% of the participants in Study 1 were White), which may have introduced potential bias into the results. The overrepresentation of White individuals could have influenced the findings by reflecting a stronger tendency toward anti-immigrant attitudes among White participants, rather than a purely narcissism-driven effect. This raises the possibility that the observed patterns may not be entirely generalizable across racial and ethnic groups. Given that previous research has shown that White individuals tend to exhibit more negative attitudes toward immigrants (Smeltz et al., 2023), the high proportion of White participants in the sample may have strengthened the relationship between narcissism and anti-immigrant sentiment. To determine whether these patterns extend beyond White individuals, future studies should include more racially and ethnically diverse samples. Additionally, examining samples with a broader range of demographic characteristics—such as age, socioeconomic status, and educational background—would help assess the extent to which these findings generalize beyond the current samples.

Given the results of this research, there are several avenues for future investigation. One potential direction is to further explore how narcissistic individuals’ attitudes toward outgroups, such as immigrants, may vary depending on contextual factors, such as economic threat or political climate. For instance, previous research has suggested that intergroup hostility is more likely to occur in situations where individuals feel their social status or resources are threatened (Duckitt et al., 2002). Research exploring whether narcissistic individuals become more prejudiced in times of perceived social or economic instability would provide important insights into the situational determinants of narcissism’s connections with anti-immigrant attitudes. Additionally, future research could explore how interventions targeting underlying social worldviews—such as reframing how narcissistic individuals think about the social environment to reduce perceived competition—might mitigate the negative effects of narcissism for intergroup relations.

## 6. Conclusions

The present research contributes to a deeper understanding of the complex connections between narcissistic traits and anti-immigrant attitudes. By examining the mediating roles of social worldviews and ideological attitudes, we provide valuable insights into how narcissism might be connected with prejudice through perceptual lenses such as the competitive social worldview and the adoption of ideological attitudes such as RWA and SDO. These findings underscore the importance of considering the multifaceted nature of narcissism in the context of prejudice, suggesting that different narcissistic traits may differ in their connections with anti-immigrant attitudes. As such, these studies not only expand on existing literature but also highlight the potential for future research to further explore these relationships and their implications for social and political attitudes. Ultimately, the findings suggest that addressing the connections between narcissism and prejudice may offer useful pathways for interventions aimed at reducing anti-immigrant sentiment and promoting more inclusive social attitudes.

## Figures and Tables

**Table 1:** Study 1: Intercorrelations and descriptive statistics.

	1	2	3	4	5	6	7	8
1. Extraverted Narcissism	—							
2. Antagonistic Narcissism	0.46 ***	—						
3. Neurotic Narcissism	−0.13 *	−0.18 ***	—					
4. Dangerous Social Worldview	0.11 *	0.07	0.14 **	—				
5. Competitive Social Worldview	0.14 **	0.57 ***	−0.20 ***	0.19 ***	—			
6. Right-Wing Authoritarianism	0.19 ***	0.24 ***	−0.24 ***	0.15 **	0.31 ***	—		
7. Social Dominance Orientation	0.14 **	0.48 ***	−0.26 ***	0.00	0.58 ***	0.60 ***	—	
8. Anti-Immigrant Attitudes	0.01	0.35 ***	−0.19 ***	−0.06	0.38 ***	0.43 ***	0.54 ***	—
Mean	3.18	2.18	3.35	4.27	3.02	−0.79	2.73	1.71
Standard Deviation	0.66	0.57	0.73	0.89	0.83	1.11	1.05	1.07

* *p* < 0.05; ** *p* < 0.01; *** *p* < 0.001.

**Table 2:** Study 2: Intercorrelations and descriptive statistics.

	1	2	3	4	5	6	7	8
1. Extraverted Narcissism	—							
2. Antagonistic Narcissism	0.59 **	—						
3. Neurotic Narcissism	−0.11 *	−0.03	—					
4. Dangerous Social Worldview	0.12 **	−0.15 **	0.09 *	—				
5. Competitive Social Worldview	0.34 **	0.70 **	−0.03	0.26 **	—			
6. Right-Wing Authoritarianism	0.25 **	0.30 **	−0.12 **	0.52 **	0.32 **	—		
7. Social Dominance Orientation	0.33 **	0.57 **	−0.12 **	0.21 **	0.59 **	0.51 **	—	
8. Anti-Immigrant Attitudes	0.39 **	0.68 **	−0.13 **	0.15 **	0.54 **	0.44 **	0.60 **	—
Mean	3.08	2.41	3.12	3.96	3.05	−0.66	2.80	2.32
Standard Deviation	0.74	0.76	0.71	1.01	0.91	1.43	1.18	1.69

* *p* < 0.01; ** *p* < 0.001.

**Table 3:** Study 3: Intercorrelations and descriptive statistics.

	1	2	3	4	5	6	7	8
1. Extraverted Narcissism	—							
2. Antagonistic Narcissism	0.44 ***	—						
3. Neurotic Narcissism	−0.04	0.08 *	—					
4. Dangerous Social Worldview	0.12 **	0.15 ***	0.08 *	—				
5. Competitive Social Worldview	0.16 ***	0.52 ***	0.05	0.40 ***	—			
6. Right-Wing Authoritarianism	−0.03	0.11 **	0.02	0.26 ***	0.24 ***	—		
7. Social Dominance Orientation	0.07 *	0.37 ***	−0.03	00.09*	0.42 ***	0.27 ***	—	
8. Anti-Immigrant Attitudes	0.05	0.28 ***	−0.08 *	0.16 ***	0.38 ***	0.47 ***	0.35 ***	—
Mean	3.34	2.36	3.07	4.08	2.89	−0.29	2.91	1.92
Standard Deviation	0.58	0.43	0.66	0.92	0.74	1.06	0.87	1.04

* *p* < 0.05; ** *p* < 0.01; *** *p* < 0.001.

## Data Availability

The original data presented in the study are openly available from the Open Science Framework (OSF) at https://osf.io/fv5cx/ (accessed on 5 February 2025).
